# Inspections in America and Germany

**Published:** 1920-05-08

**Authors:** 


### Inspections in America and Germany.

With the time and space available it is not possible to do more than mention the chief facts of his periodic visits to America. The first principal date, is that of his early visit in 1890, when he took pains to see a number of institutions, and had to travel long distances in consequence. All he saw, and according to his custom his experiences were documented, stimulated his desire to go again. A later visit was paid in 1895, and another ten years later. He crossed the Atlantic for the last time in 1916, when the passage was prolonged for every boat in consequence of the German submarines in the Atlantic. This record would defeat its own end if it seemed to aim at an impossible completeness. Let it suffice to say that it was in America that Sir Henry made his friendship with the late Dr. John S. Billings, of the Johns Hopkins Hospital, Baltimore, who is still remembered for his work for the wounded during the American Civil War, and for the unwearied pains

**America and Germany**—(*continued*).

which he took to collect the volumes and produce the Index Catalogue of the New York Public Library. This Index Catalogue has grown greatly, and the principles on which the indexing was designed have governed the work throughout. Their mutual interest in method and statistics, which became almost a theory in favour of a mechanical memory, united the two men, who sought each other's help, and made great and willing inroads on each other's time accordingly. Dr. Billings was advised on the plans of the Johns Hopkins University, and such advice as was given involved plenty of hard work. This perhaps is a typical example of what was learnt in America; though not only American construction but also its classified system of pay wards, the absence of anything corresponding to an English Cottage Hospital, the willingness in American institutions to answer questions and exchange information, were generally observed, not only for their own interest, but for the sake of the contrast that they provided to English institutional life. Neither system was held in all respects the superior, and, just as there is now a cry for something in America of the size and character of a cottage hospital, so, the traveller thought, the American pay-ward on a graduated system might with advantage be tried, if suitably modified here. Are not circumstances on the side of this advocacy? Since the war there has been occasion to mention his Reports upon German Hospitals after a visit to Germany in 1912-13. They admitted the excellence of the equipment, the beautifully kept gardens, the mechanical side of the work, the laborious devotion to science, and so forth. But the Reports complained that this spirit had its inhumanity; any who may like to test the value of the criticism may remember that German scientific methods were very much in fashion at the time.

